# The use of the transition cost accounting system in health services research

**DOI:** 10.1186/1478-7547-5-11

**Published:** 2007-08-08

**Authors:** Arik Azoulay, Nadine M Doris, Kristian B Filion, Joanna Caron, Louise Pilote, Mark J Eisenberg

**Affiliations:** 1 Divisions of Cardiology and Clinical Epidemiology, Jewish General Hospital, McGill University, Montreal, Quebec, Canada; 2 Divisions of Clinical Epidemiology and Internal Medicine, Montreal General Hospital, McGill University, Montreal, Quebec, Canada

## Abstract

The Transition cost accounting system integrates clinical, resource utilization, and financial information and is currently being used by several hospitals in Canada and the United States to calculate the costs of patient care. Our objectives were to review the use of hospital-based cost accounting systems to measure costs of treatment and discuss potential use of the Transition cost accounting system in health services research. Such systems provide internal reports to administrators for formulating major policies and strategic plans for future activities. Our review suggests that the Transition cost accounting information system may useful for estimating in-hospital costs of treatment.

## Background

Recent technological innovations and the increasing complexity of medical care in Canada and the United States (US.) have emphasized the need for a cost-effective approach to the care of patients admitted to health care institutions. National-level data indicate that between 1960 and 2002 the proportion of gross domestic product spent on health care increased from 5.5% to 9.9% in Canada and from 5.3% to 14.6% in the US. [1,2]. As such, hospital administrators are beginning to take interest in implementing cost accounting information systems, or software systems for cost accounting. The Transition system, for example, is a commercially available hospital cost accounting system currently used in Canada and the US. (Eclipsys Solutions Corporation, Boca Raton, FL). This system integrates large volumes of patient-level clinical and financial information into a single database [3]. The Transition system methodology entails a certain amount of complexity, and several sources of measurement error may compromise the accuracy of its measurements. In addition, inter-hospital variations in cost accounting practices may affect the accuracy of cost estimates when using the Transition system to compare costs between hospitals. Our objectives are to review the ability of Transitions system to measure costs of treatment, to discuss the system's methodology, and to describe the system's potential use in health services research.

## Traditional methods for estimating in-hospital costs of treatment

Traditionally, sources of information for estimating costs of treatment in Canadian and US. hospitals included generic per diem costs, specialty per diem costs, and costs per weighted case [4]. *Generic per diem costs *are daily dollar rates that represent the average cost of one hospitalization day irrespective of the patient's medical condition. *Specialty per diem costs *are daily dollar rates established for specific hospital departments and represent the average cost of hospitalization in specific departments. *Costs per weighted case *capture the cost of hospitalization of a patient in a specific condition and are usually classified according to clinical diagnoses. In spite of the availability of such estimates in many hospitals, these rates represent measurements that may be inaccurate for research purposes. Costs per weighted case assume that each patient consumes the same mix of services, and inter-patient variability is not captured [5]. When used as a measure of efficiency, costs per weighted case assume that the outcomes of hospital services (e.g., clinical outcomes, patient satisfaction, accessibility, quality of care) are constant and generally equivalent across institutions and over time [6]. The costs per weighted case method is therefore analogous to costing identical units on an assembly line [5].

A more accurate method for assessing the costs of health care services is that of "top-down" costing. This method is largely used by US. hospitals and involves breaking down department expenditures to obtain procedure-level costs [7]. The most prevalent top-down costing approach is the ratio of cost to charge (RCC) method. This method estimates procedure-level costs by computing an overall ratio of departmental aggregate costs to charges and applying this ratio for individual procedures and services. However, the RCC method has several limitations. First, costs derived through this method are based on aggregate information and may not accurately reflect the actual costs of a particular procedure provided within the department [7]. Second, charges are set on the basis of a variety of internal and external factors and do not necessarily maintain a constant relationship with costs (e.g., discounts) [7]. Finally, the RCC is not applicable to hospitals in socialized health care systems. In Canada, for example, hospitals do not charge third-party payers for the treatment of individual patients; therefore charge data are not available in Canada.

Another method for estimating costs of in-hospital treatment is that of "bottom-up" costing. Bottom-up costing is used partially in Australia and as a standard in Germany [8]. This costing method is more precise since the expenses associated with the treatment of any patient can be generated based on an accurate clinical cost recording system. Similarly, "micro costing" involves identifying all of the resources used in patient care, assigning costs to each resource used, and multiplying the resources used by the estimated unit costs to obtain a measure of total cost of treating a patient [4]. Although this method provides accurate cost estimates, the time and costs involved in identifying resource utilization for every patient are excessive. Therefore, analysis using micro costing is impractical for studies involving large numbers of patients.

A practical and potentially accurate method that hospitals have started to adopt to estimate costs is that of hospital cost accounting systems. Hospital cost accounting systems are software systems that integrate resource utilization and financial data already recorded in other hospital information system databases. These databases include the hospital Billing System, Payroll System, General Ledger System, and from individual departments' resource utilization databases [9]. The use of hospital-based cost accounting systems is similar to micro costing in that both methods collect data on a patient-level basis. This is important because few statistical analyses may be completed with aggregate data or generic estimates. However, unlike micro-costing, cost accounting systems use automated data collection, which allows for the collection of data for a larger number of patients and over a longer time period. As such, analysis studies involving large numbers of patients are practical using data extracted from cost accounting systems.

## The Transition cost accounting system framework

The Transition system framework views hospital activity as a three-stage process [3]. In the first stage, procedures and services provided to the patient are converted into intermediate products. In the second stage, the products are grouped to produce individual patient cases. In the third stage, patient cases are grouped to form groups of patients with a common characteristic such as a similar clinical diagnosis.

Using the Transition system software, detailed patient-level demographic, clinical, resource utilization, and cost data are integrated into a single database. For each patient, demographic and clinical data are extracted from the hospital Medical Records system and transferred into the Transition system data warehouse. These data include information from the patient's discharge summary such as the length of hospital stay, primary and secondary clinical diagnoses, and principal and secondary procedures. Unit costs are then associated with individual products and services used in the treatment of a patient, and the aggregate of these costs represents the patient's total costs of treatment within the hospital.

## The Transition system's costing methodology

An understanding of the Transition System methodology is important in order to assess the accuracy of the cost estimates provided by the system. In addition, understanding the methodology is important in order to recognize how the system can be used to undertake cost studies in health services research. The following is a detailed description of the 6-step Transition system methodology used to estimate total unit costs of products and services used in in-hospital patient care [3].

**Figure 1:**
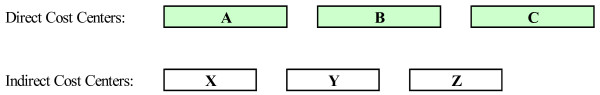
Step 1: Classification of departments as direct or indirect cost centers.

In the first step of the Transition system methodology, hospital departments are categorized as either direct or indirect cost centers (Figure 1). Each department incurs expenses that are directly or indirectly related to providing medical services. *Direct cost centers *are patient care departments (e.g., radiology, operating room) that directly provide services to patients, and the costs incurred by these departments are called *direct costs*. *Indirect cost centers *are hospital overhead departments (e.g., administration, housekeeping), and the costs incurred by these departments are called *indirect costs*.

**Figure 2:**
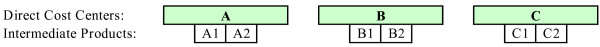
Step 2: Identification of department level intermediate products.

In the second step, intermediate products are formed by grouping the services and procedures provided in the patient care departments (Figure 2). *Intermediate products *are department specific and may represent either a product (e.g., catheter, medication) or a service (e.g., nursing care, x-ray) or a combination of products and services used in patient care. One example of an intermediate product that is a product includes the medications provided by the pharmacy or a hospital gown from central supplier. Intermediate products that are services include a cardiopulmonary resuscitation in the emergency department or nursing care in the intensive care unit. Examples of intermediate products that combine products and services include a coronary angioplasty in the cardiac catheterization laboratory or a chest x-ray in the department of radiology.

Hospital and department managers have complete discretion in the definition of intermediate products since intermediate products defined in the Transition system must directly mirror the activities that are recorded on patient records. Hospitals use the definitions of the intermediate products defined in their patient care systems (e.g., lab result reporting system, diagnostic imaging system, nursing database) and can therefore identify the specific patient for whom each intermediate product was recorded. Typically, US. hospitals use the definitions from their billing system for intermediate product identification.

**Figure 3:**
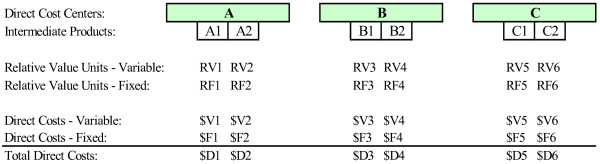
Step 3: Estimation of direct costs of intermediate products.

In the third step, estimations of the relative direct costs of each department's intermediate products are derived (Figure 3). Direct costs include direct labor costs and direct materials costs. *Direct labor costs *are costs related to the actual labor of individual employees within the department (e.g., salaries and fringe benefits of nurses and technicians). *Direct materials costs *are all department-level non-labor costs that become part of the patient care process (e.g., pharmaceutical products, supplies). Direct costs may be classified as fixed or variable costs depending on their responsiveness to fluctuations in volume.

In order to estimate an intermediate product's direct costs, the weighted procedure method is employed. With this method, every intermediate product is assigned a number of relative value units (RVUs). The RVU represents the intermediate product's estimated consumption of equipment, supplies, and personnel time. In other words, RVUs are an expression of the relative direct costs of one intermediate product to another within a given patient care department [10].

RVUs can be calculated based on nationally available standards or based on actual cost data. In Canada, for example, labour RVUs are nationally or provincially engineered time values which are mandated by the Ministry of Health. Alternatively, labor RVUs are also obtained based on actual minutes of time as recorded during the performance of the procedure. Supplies use an RVU calculated based on the average actual cost for the supplies normally used to perform the activity. RVUs for prescribed medications are based on the actual costs to the department.

The fixed and variable direct costs of a single RVU can be calculated once RVUs have been allocated to every intermediate product within a department. Costs are calculated by dividing the department's total fixed and variable costs, respectively, by the department's total number of RVUs used throughout a period. The variable and fixed direct cost of each intermediate product can subsequently be estimated by multiplying the intermediate product's assigned variable and fixed RVUs by the cost of a single RVU. For example, a department with 100 RVUs and total direct and indirect costs of $20,000 and $10,000, respectively, will allocate $200 of direct costs and $100 of indirect costs for each RVU. An intermediate product to which the department assigned 3 RVUs will therefore represent a total cost of $900, or $600 in direct costs and $300 in indirect costs.

**Figure 4:**
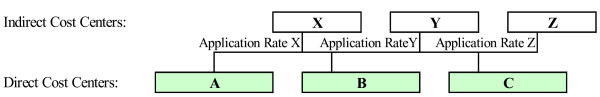
Step 4: Identification of application rates for allocating indirect costs.

In the fourth step of the Transition methodology, application rates are identified in order to assign indirect costs to direct cost centers (Figure 4). A base for allocation must first be determined for each type of indirect cost, or cost pool. A *cost pool *is any grouping of costs to be allocated, and a *base*, or cost driver, is a criterion upon which the allocation is to be made [11]. Using the cost pool and base, an *application rate *is determined to allocate the total costs of an indirect cost center to a number of direct cost centers. For example, the total costs of a hospital's housekeeping services are usually assigned according to square footage. An application rate would then be determined by dividing total hospital housekeeping costs by total hospital square footage. A rate of $0.50/square foot, for example, indicates that each department will be allocated $0.50 per square foot for housekeeping services provided. While for some indirect cost pools, such as housekeeping, a fairly accurate and plausible allocation basis can be found (e.g., square footage), many other indirect costs are much more difficult to allocate in a plausible way. The costs of central administration are one example of indirect costs that are difficult to distribute.

**Figure 5:**
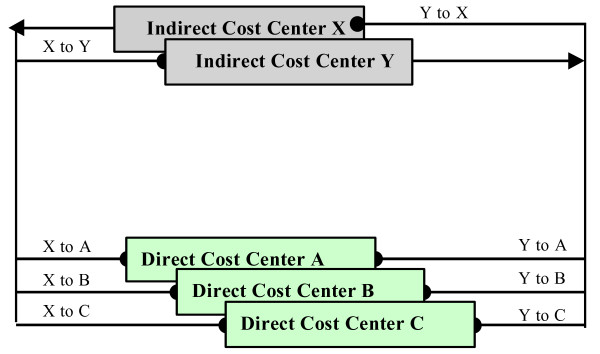
Step 5: Allocation of indirect costs to direct cost centers.

In the fifth step, indirect cost centers are allocated using an allocation algorithm (Figure 5). The step-down method is the commonly used method for allocating these indirect costs. Under this method, indirect cost centers are ranked in terms of decreasing amounts of service offered to other centers, and their costs are allocated one at a time in descending order. An indirect cost center is deemed "closed", once the cost of the indirect cost center have been determined, no other cost center can assign costs to it, and therefore, in the analysis, there remains one less center [10]. The assumption of a one-way service between departments works well enough for financial reporting and in some cases represents the flow of the use of services quite well [12]. However, the step-down method may become less accurate as the interactions among service departments become more important. Consequently, the user may choose to use another allocation algorithm such as the reciprocal allocation method. This method is conceptually appealing because it recognizes the simultaneous interaction of service departments rather than the somewhat arbitrary, one directional relationship the step-down method assumes [12].

**Figure 6:**
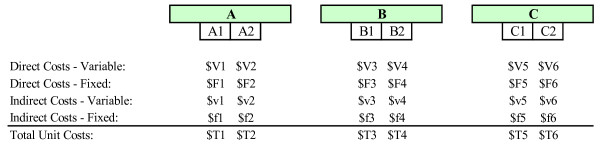
Step 6: Estimation of indirect costs and total unit costs of intermediate products.

In the sixth step, the indirect costs that were previously assigned to patient care departments are allocated, within each department, to intermediate products (Figure 6). The RVUs previously assigned to each intermediate product are used to allocate intermediate products. First, the indirect cost of a single RVU is estimated by dividing the total indirect costs assigned to the department by the department's total number of RVUs. The indirect cost of each intermediate product is then estimated by multiplying the intermediate product's assigned number of RVUs by the indirect cost of a single RVU. Once indirect costs have been assigned to individual intermediate products, the user is able to estimate the total unit costs of intermediate products by adding the product's direct costs (fixed and variable) and indirect costs.

## Potential sources of error in determination of unit costs

Using the Transition system methodology, there are several potential sources of measurement error when estimating the unit costs of products and services. Bias may occur when intermediate products are identified and when direct and indirect dollar values are assigned to each intermediate product. In the second step of the Transition system methodology, procedures and services provided in patient care departments are selected and grouped into discrete intermediate products. The identification of the intermediate products at each department is generally based on the assumption that a relatively small number of procedures and services make up a high percentage of the department's costs. Department managers generally follow the "80/20 rule" thereby identifying the 20% of a department's products and services that account for 80% of its costs [13]. Nonetheless, this ratio is arbitrary and can vary among different hospitals and even among different departments within a single hospital. Measurement bias may occur if department managers identify only a portion of the total number of intermediate products used in their department. The direct costs (incurred by the department) and the indirect costs (allocated to the department) are therefore assigned to the selected intermediate products identified by the department manager. Consequently, the unit costs of individual intermediate products can be overestimated.

Measurement bias may also arise due to misclassification of fixed and variable costs. In the third step of the Transition system methodology, the RVUs of individual intermediate products are estimated based on the total resources consumed when the product or service is used in patient care. The potential for measurement bias arises because a portion of the product's consumption of fixed costs may be considered as variable costs and, conversely, variable costs related to the product may be considered as fixed costs. Depending on the circumstances, the variable and fixed costs of a given intermediate product may be either overestimated or underestimated.

Another potential source of measurement bias arise from the incorrect allocation of cost. For example, in the fourth step of the Transition system methodology, application rates are identified in order to allocate indirect costs to direct cost centers. Ideally, indirect costs should be allocated based on cost drivers that cause the minimum amount of distortion in cost allocation. Cost drivers used for allocating indirect costs typically include square footage, pounds of laundry, patient days, or total direct costs incurred by the department. The potential for measurement bias arises here because financial managers may allocate indirect costs to direct cost centers based on imperfect cost drivers. For example, indirect costs may be allocated to direct cost centers based on the total direct costs incurred by the patient care departments. In such cases, a given patient care department may be allocated a bigger or smaller portion of indirect costs than is truly appropriate. Consequently, indirect costs of individual intermediate products will be overestimated or underestimated depending on the circumstances.

Incorrect allocation of algorithms is another potential source of measurement bias in costing accounting systems. In the fifth step of the Transition system's analysis, an allocation algorithm is used in order to allocate indirect costs to direct and indirect cost centers. A common algorithm for allocating indirect costs to direct cost centers is the step-down method. Using this method, indirect cost centers are ranked in terms of decreasing amounts of service offered to other centers, and their costs are allocated one at a time in descending order. The order of allocation can have a significant impact on which department ultimately bears the costs of the organization [10]. The potential for measurement bias may arise if the hierarchy of indirect cost centers is not accurate. Consequently, the costs allocated to service departments may be overestimated or underestimated, and indirect costs allocated to intermediate products may also be incorrectly estimated.

There are also numerous situations in which service departments service or interact with each other simultaneously [12]. For example, personnel from housekeeping and maintenance also service administration offices. In general, the step-down method will not be sufficiently accurate when extensive interactions exist among service departments. This is where the reciprocal method becomes valuable. Under the reciprocal allocation method, the total amount of a particular indirect cost center's cost that is allocated is affected by the reciprocity of services that each indirect cost center provides the other indirect cost centers.

## Assessing the accuracy of Transition's financial, clinical, and resource utilization information

Transition's various applications access the same central, single database, or Transition data warehouse. The system thus offers a comprehensive clinical and financial database for both hospital inpatients and outpatients. Raw data are transferred into Transition from "feeder systems" such as the hospital's General Ledger (resource utilization data), Medical Records (demographic and clinical data), and Billing systems (financial data). The assessment of the accuracy of Transition's financial, clinical, and resource utilization information must therefore consider two major sources of measurement error. The first concerns the accuracy of the original data, i.e. the extent to which the data in the feeder systems are recorded precisely and without bias. *Inter-observer variation *results from inconsistencies between different individuals recording the data, and *intra-observer variations *results from inconsistencies by the same observer on different occasions [14]. The second source of measurement error concerns the accuracy of Transition's data, for example, the extent to which the information in Transition's data warehouse accurately estimates the information that was originally recorded in the feeder systems.

To examine the accuracy of the data transferred from the feeder systems, we examined the results of an audit performed at one US. hospital. Our previous study suggests that the Transition data warehouse is likely to contain information that is as accurate as that recorded in its feeder systems. This analysis sought to assess the magnitude of discrepancies between information available in the Transition data warehouse and its feeder systems. At this US. hospital, utilization and financial data were compared between the Transition system and its three major feeder systems: the Medical Records system, the Billing System, and the General Ledger system. Documentation from the auditing of the feeder systems at this US. hospital suggests that the accuracy of data is not likely to be compromised when information is transferred from one system to another.

The standardized use of clinical information systems in Canadian and US. hospitals suggests that their Medical Records systems are also likely to contain data that are accurate [15]. Using the clinical information recorded in the Medical Records system, hospitals classify patients into distinct diagnosis-related groups (DRGs). This classification is based on the patient's demographic and clinical characteristics and course of treatment in the hospital. The accurate classification of DRGs is important for US. hospitals because patient-level reimbursement is based on DRG-specific charges that are negotiated with private and public third-party payers. Similarly, the accurate classification of patients into DRGs is important for Canadian hospitals in the negotiation of yearly global operating budgets with provincial governments. In addition, the data utilized by the Canadian Institute for Health Information (CIHI) Discharge Abstract Database (DAD) are the same data extracted from resource and cost accounting systems. In order to ensure the validity of this data, the CIHI established a data quality program which includes the implementation and ongoing examination of a corporate Data Quality Framework [16].

## Inter-hospital cost comparisons

Differences in the classification of direct and indirect cost centers may arise if hospitals outsource patient care services to independent contractors. Consider two hospitals, for example, where one hospital provides on-site computed tomographic scans while the other hospital purchases these services from an outside supplier. For the first hospital, the costs related to the scans (e.g., supplies and equipment) will be incurred by the radiology department, a direct cost center and will be assigned to individual patients as direct costs. For the second hospital, however, the costs of the scans will be assigned to an indirect cost center and subsequently allocated to direct cost centers. In such cases, costs defined as direct in one organization may be categorized as indirect in another [17]. Misclassification bias in cost estimates is likely to be differential, and differences in costs may be either overestimated or underestimated.

In addition, variations in RVU estimates can also lead to information bias. The weighted procedure method for estimating the RVUs is one method for correcting problem, however, it is a costly and time-consuming endeavor. In fact, this procedure is so costly that most department managers use industry standards rather than computing the RVUs themselves [10]. The use of industry-wide standards, however, assumes that all hospitals are exactly the same, and that the resource consumption for each intermediate product relative to all other products is the same across all hospitals. This assumption is highly unlikely given that hospitals generally differ in the resources used in delivering care. Differences in costs between two hospitals are not likely to be biased if RVUs are calculated by the department managers of each of the two hospitals. In fact, this ideal scenario will yield the most accurate cost estimates.

Differences in the allocation of indirect cost are another source from which information bias may occur. A careful examination of hospital cost centers may disclose the presence of unusual indirect costs at a particular hospital. Costs related to research activities, affiliated medical schools, bad debts, interest expense, or bond interest, for example, may be present in one hospital's indirect cost pool but not in another. The potential for bias may arise if such expenses are not removed from the cost analysis. In such cases, the misclassification is likely to be differential, and the overall difference in costs between the two hospitals will be overestimated. Other potential sources of differential cost misclassification arise when hospitals use different application rates and allocation algorithms to allocate indirect costs to direct cost centers.

Once there is an understanding of the Transition System's methodology, the system can be applied in multi-center studies. For example, the Transition system has been used to examine the cost of treatment of abdominal aortic aneurysm (AAA) repair in Canada and the US. [18]. This same cohort was also used to examine the cost of hospital readmissions following AAA repair [19]. In addition, the transition system has been applied to determine in-hospital cost of total hip arthroplasty and coronary artery bypass graft (CABG) surgery in Canada and the US. [20,21] and the impact of age [22], sex [23], and non-elective status [24] on hospital course and cost among patients undergoing CABG.

## Future directions for health services researchers

Several published studies have previously used the Transition cost accounting system to estimate the costs of health care services at individual hospitals [11,25-30]. In general, the accuracy of the Transition system's measurements relies primarily on the accuracy of the data extracted from the hospital's feeder systems and on the hospital's specific choices for estimating unit costs of products and services with the Transition software. Further studies are needed in order to assess the errors compromising the measurement of costs on the Transition system's cost estimates.

Despite its limitations the Transition cost accounting system may be an important tool for health services research for several reasons. First, patient-level resource utilization information can be used to examine and compare patient management techniques between different physicians, hospitals, and health care systems. Second, unit costs of products and services estimated by the Transition software can be used in the economic evaluation of hospital-based health care interventions. This information may be especially useful for cost of illness studies and for the evaluation of alternative treatment programs through the undertaking of cost effectiveness, cost benefit, and cost utility analyses [31]. Third, the availability of detailed demographic and clinical information in the Transition system data warehouse can be used to identify patient characteristics that are associated with increased costs of treatment.

## Conclusion

The potential use of the Transition system in health services research largely depends on the accuracy of the cost estimates provided by the software. Due to the possibility for error in measuring costs, it is crucial to understand the Transition system methodology for estimating unit costs of products and services. The Transition system has the potential to provide internal reports to administrators for use in formulating major policies and strategic plans for future activities. Although the primary use of hospital cost accounting systems is for internal management purposes, data extracted from these systems may be useful for conducting cost of illness studies and health services research.

## Authors' contributions

AA conceived of the manuscript, contributed to the study design and coordination, and drafted and revised the manuscript. NMD, KBF, and JC revised the manuscript. LP and MJE contributed to the study design and manuscript revisions. All authors read and approved the final manuscript.
